# Total Laparoscopic Hysterectomy with Prior Uterine Artery Ligation at Its Origin

**DOI:** 10.1155/2014/420926

**Published:** 2014-11-19

**Authors:** Vidyashree Ganesh Poojari, Vidya Vishwanath Bhat, Ravishankar Bhat

**Affiliations:** ^1^Department of OBG, Radhakrishna Multispeciality Hospital, Bangalore, Karnataka, India; ^2^Department of General Surgery, Radhakrishna Multispeciality Hospital, Bangalore, Karnataka, India

## Abstract

We compared the duration of surgery, blood loss, and complications between patients in whom both uterine arteries were ligated at the beginning of total laparoscopic hysterectomy (TLH) and patients in whom ligation was done after cornual pedicle. Using a prospective study in a gynecologic laparoscopic center, a total of 52 women who underwent TLH from June 2013 to January 2014 were assigned into two groups. In group A, uterine arteries were ligated after the cornual pedicles as done conventionally. In group B, TLH was done by ligating both uterine arteries at the beginning of the procedure. All the other pedicles were desiccated using harmonic scalpel or bipolar diathermy. Uterus with cervix was removed vaginally or by morcellation. The indication for TLH was predominantly dysfunctional uterine bleeding and myomas in both groups. In group A, the average duration of surgery was 71 minutes, when compared to 60 minutes in group B (*P* < 0.001). In group A, the total blood loss was 70 mL, when compared to 43#x2009;mL in group B (*P* value < 0.001). There were no major complications in both groups. To conclude, prior uterine artery ligation at its origin during TLH reduces the blood loss and surgical duration as well as the complications during surgery.

## 1. Introduction

Hysterectomy is a common gynecological procedure worldwide for benign uterine disease. Traditionally, this has been via the abdominal or vaginal routes [1]. In the present era, hysterectomies are undertaken using minimal access techniques. Total laparoscopic hysterectomy (TLH) is performed entirely by the laparoscopic route, including closure of the vaginal vault, with the uterus being removed vaginally or by morcellation [2].

Today, lap hysterectomy is a safe and feasible technique to manage benign uterine pathology as it offers minimal postoperative discomfort, shorter hospital stay, rapid convalescence, and early return to the activities of daily living [3]. Considerable technical advances in this procedure have occurred during the last few years.

In our study, we have modified the steps and started with the ligation of the uterine artery at its origin from the internal iliac artery on both sides causing transient uterine ischemia as most blood enters the uterus through these vessels especially its ascending branch [4]. The hypothesis of this study proposes that, soon after occlusion, blood within the myometrium clots and the myometrium becomes hypoxic.

The aim of this study was to compare conventional TLH to prior uterine artery ligation at its origin.

## 2. Materials and Methods

It was a prospective randomized controlled study conducted from June 2013 to January 2014. Total of 52 cases were included in the study of which 26 underwent conventional total laparoscopic hysterectomy and another 26, underwent uterine artery ligation at its origin prior to total laparoscopic hysterectomy. Ethical clearance and informed consent were obtained for the study.

All patients underwent preoperative evaluation. Patients were kept nil by mouth 12 hours before the procedure and no bowel preparation was done prior to surgery. Catheterization of the urinary bladder was done preoperatively. Antibiotic prophylaxis was given to all patients included in the study. Compression devices were given to all patients for prophylaxis against possible thromboembolic episodes. Subcutaneous low molecular weight heparin was given postoperatively in obese patients.

## 3. Surgical Technique

**Figure 1:**
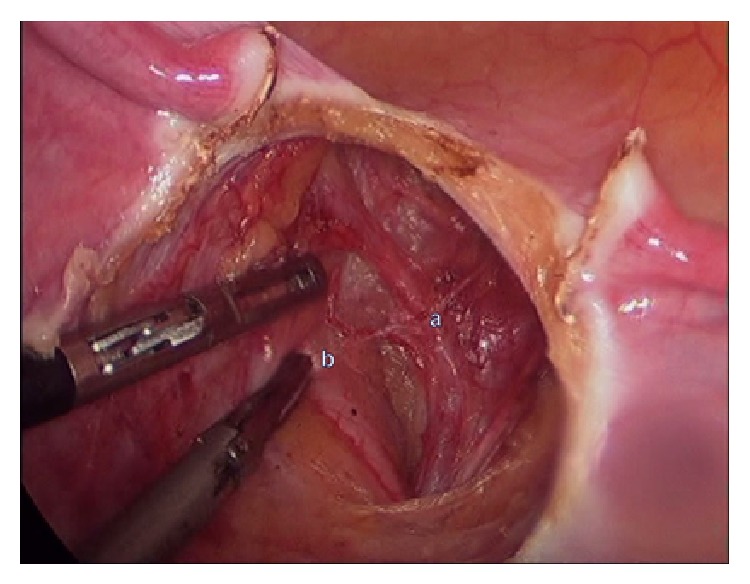
Identifying (a) uterine artery and (b) ureter by lateral dissection.

**Figure 2:**
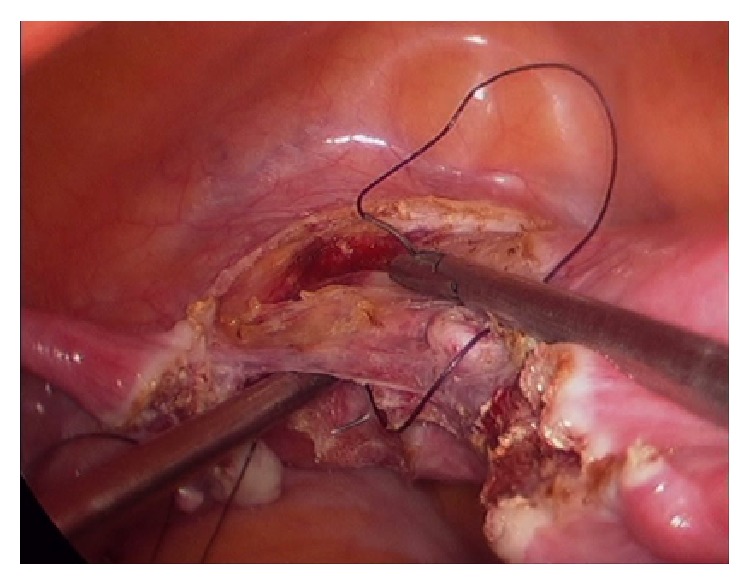
Prior ligation of uterine vessels.

Under general anesthesia, the patient was placed in modified lithotomy position. A Veress needle is inserted at the umbilicus or supraumbilical site depending on the size of the uterus and abdomen is insufflated with carbon dioxide at initial pressure of 20 mm Hg and maintenance at 15 mm Hg. A 10 mm trocar is inserted blindly and 10 mm telescope is introduced through this port. Uterus and the adnexa were visualized. Three additional 5mm ports are introduced: one along the left spinoumbilical line at the junction of medial 2/3rd and lateral 1/3rd, second port at right angles to the previous port two ports, and a third 5 mm port placed around 2 cm below and to the right of umbilicus. The entire abdomen is surveyed before starting the procedure. The size of the uterus, presence of myomas, and adnexa and course of ureters are visualized. Manipulation of the uterus was done with a 5 mm myoma spiral laparoscopically. No vaginal manipulators were used. Uterine artery was dissected by a lateral approach. A window was created in the broad ligament, close to the uterine vessels. The ascending branch of the uterine artery is identified close to the isthmus (Figure 1). The uterine vessels are ligated at this level close to the uterus using 1-0 delayed absorbable suture material or coagulated using bipolar diathermy (Figure 2). The uterine artery is not sutured away from the uterus as the ureters cross beneath them at that level. Dissecting the uterovesical fold and pushing the bladder down move the ureters laterally and decrease the risk of including them in the suture. The vasculature of the uterus is thus secured and this is evidenced by the color change in the fundus, which becomes pale. The cornual pedicles on one side are then desiccated and cut either using bipolar diathermy or the harmonic ultracision. The ligated uterine pedicles are cut. The uterosacral and cardinal ligaments are desiccated and cut. The position of the myoma spiral is then changed so that the opposite side pedicles can be taken care of.

If both ovaries need to be removed, the infundibulopelvic ligaments are desiccated and cut. Now a vaginal cuff is introduced through the vagina to identify the vault and the anterior lip of cervix held with a tenaculum. Vault cut laparoscopically using monopolar hook and the specimen is detached completely. The uterus with cervix is delivered vaginally if small. In case of large uteri, the specimen can be retrieved by morcellation through abdominal port. We prefer to use the contralateral ports for suturing. The right midquadrant port and the left lower quadrant port are ergonomically apt for suturing. The vaginal vault is sutured with number 1 delayed absorbable suture (vicryl). Ports closed using staples. The total blood loss is calculated from the suction apparatus. The blood in the suction tube is also measured to give the accurate value. No irrigation is used throughout the procedure until the calculation of the total blood loss. Peritoneal lavage is given with normal saline solution and 500 mL of normal saline is left in the peritoneal cavity. The catheter is removed after 6 hours and liquid diet started after peristalsis is established. The patient is discharged the following day and called for follow-up after 7 days.

## 4. Results

52 cases were included in the study, of which 26 underwent conventional TLH (Group A) and another 26 underwent TLH with prior uterine artery ligation at its origin (Group B). Sociodemographic data were similar in both groups. The main symptoms of patients were similar in both groups, with the predominant being menorrhagia (74.9% in group A and 74.1% in group B). Indication for surgery also revealed similar results in both groups as shown in Table 1. In group A, 72.9% of women had previous normal delivery and 27.1% had previous cesarean section and in group B 68% had previous normal delivery and 22% had previous cesarean section. In patients with previous cesarean section, because of dense bladder adhesions in some cases, there were difficulties in dissection.

Clinical size of the uterus ranged from 10 weeks to 22 weeks in both groups. The hemoglobin levels in all patients of both groups were above 9 g/dL. None of them required preoperative blood transfusion. In group A, 64.6% of specimens were retrieved vaginally and 35.4% of specimens were morcellated and retrieved. In group B, 68.4% of specimens were removed vaginally and 31.6% were retrieved by morcellation.

Total duration of surgery and blood loss in both groups were compared (Table 2). In group A, the average duration of surgery was 71 minutes. In group B, the average duration of surgery was 60 minutes. The comparison between the 2 groups revealed a statistically significant difference (*P* < 0.001) in duration of surgery between the 2 groups. The time taken was less in patients where the uterine artery was prior ligated.

In group A, the total blood loss was 70 mL. In group B, the total blood loss was 43 mL. The comparison between the 2 groups revealed a statistically significant difference (*P* value < 0.001). The data reveal that a significant decrease in blood loss and need for blood transfusion existed in group B where the uterine arteries were ligated before dividing the cornual structures.

There were no major complications in both of the groups. One patient in Group B with multiple fibroids and previous 2 lower segment cesarean section (LSCS) had bladder injury, detected postoperatively, and was treated conservatively with catheterization for 2 weeks.

## 5. Discussion

Total laparoscopic hysterectomy is currently accepted as an alternative to standard abdominal hysterectomy.

The vascular supply of the uterus is mainly derived from the uterine and ovarian arteries. Because most blood enters the uterus through the uterine arteries, transient uterine ischemia occurs after uterine artery ligation [5]. Bilateral uterine vessel ligation is an efficient method to obliterate the blood flow to the uterus [6].

Like most studies, we believe that the main step in hysterectomy is securing the uterine vascular pedicle [7]. Enlarged uteri allow limited access to uterine vascular pedicles depending on the size and location of myomas and may be associated with high risk of complications such as hemorrhage, ureteric injury. Prior dissection of uterine artery at its origin and ligating helps in reducing the blood supply and also lowering the risk of ureteral injury [7, 8].

To reduce the total blood loss and the duration of surgery, in this study we ligated the uterine arteries as the first step before tackling the other pedicles. In case of very large uterus, it may sometimes be difficult to dissect the ureter away from the uterine arteries properly. In such situations, coagulation of uterine vessels poses the risk of thermal damage to the ureters if they were not fully mobilized. Therefore, it is best to perform intracorporeal suturing of uterine vessels.

Our study showed that the average blood loss during the procedure is considerably reduced if the uterine vessels are primarily handled at its origin as compared to the study done by Sinha et al. [7]. The fear of ureteric injury is mostly caused by lack of familiarity with the pelvic anatomy. Once the bladder is dissected down, the ureters fall laterally and move away with the peritoneum. Hence, the risk of including the ureters in the suture is practically negligible. The risk of ureteric injuries is lower using suture compared with bipolar desiccation or staples [9].

No major complications occurred in our study. Only one patient in Group B with multiple fibroids and previous 2 LSCS had bladder injury, detected postoperatively, and was treated conservatively with catheterization for 2 weeks comparable to Sinha et al. [7].

## 6. Conclusion

Prior uterine artery ligation at its origin during TLH reduces the blood loss and surgical duration as well as the complications during surgery. As the expertise of the surgeon increases in retroperitoneal dissection, the duration of the procedure also reduces considerably.

## Figures and Tables

**Table 1:** Indications for surgery in both groups.

Diagnosis	Group A		Group B	
	No.	%	No.	%
Abnormal uterine bleeding (AUB)^*^	13	50.0	14	53.8
Endometriosis	3	11.5	3	11.5
Fibroid	10	38.4	10	38.4

Total	26	100.0	26	100.0

^*^AUB are those cases where medical management failed.

**Table 2:** Comparison of blood loss and duration of surgery between groups A and B.

Parameters	Group A	Group B	*P* value
Duration of surgery (min)	71.35 ± 5.21	60.77 ± 5.04	<0.001
Blood loss (mL)	70.96 ± 18.33	43.08 ± 5.67	<0.001
